# Lived-Experience of Women’s Well-Being in the Cyclone Shelters of Coastal Bangladesh

**DOI:** 10.1017/S1049023X2200070X

**Published:** 2022-08

**Authors:** Tazrina Jahan Chowdhury, Paul Arbon, Kristine Gebbie, Robert Muller, Mayumi Kako, Malinda Steenkamp

**Affiliations:** 1. Institute for Sustainable Futures, UTS, Sydney, Australia; 2. Torrens Resilience Initiative, Flinders University, Adelaide, Australia; 3. Hiroshima University, Higashihiroshima, Japan; 4. Department of the Premier and Cabinet, SA Government, Adelaide, Australia

**Keywords:** Bangladesh, community preparedness, disaster, well-being, women

## Abstract

Bangladesh is repeatedly threatened by tropical storms and cyclones, exposing one-third of the total population of the country. As a preparedness measure, several cyclone shelters have been constructed, yet a large proportion of the coastal population, especially women, are unwilling to use them. Existing studies have demonstrated a range of concerns that discourage women from evacuating and have explored the limitations of the shelters, but the experiences of female evacuees have not been apparent in these stories. This study explores the lived-experiences of women in the cyclone shelters of Bangladesh and discusses their health and well-being as evacuees in the shelters. Nineteen women from three extremely vulnerable districts of coastal Bangladesh were interviewed. Seven research themes were identified from the participants’ narratives using van Manen’s thematic analysis process. The most salient theme, being understood (as a woman), portrayed the quintessential image of these women, which subsequently influenced their vulnerability as evacuees. The next themes–being a woman during crisis, being in a hostile situation, being fearful, being uncertain, being faithful, and being against the odds–focused on the incidents they lived through which affected their physical and mental health and the emotions they felt as evacuees. The paper offers a deep inquiry into women’s experiences of well-being in the shelters and recognizes the significance of women’s voices to improve their experiences as evacuees.

## Introduction

Bangladesh is one of the most disaster-prone nations in the world. The country has a land area of 147,570 sq km and a high population density with a total of 160.8 million people.^1^ Because of the geographical location and flat topography, tropical cyclones and associated water surges occur frequently in the coastal belt of Bangladesh, exposing approximately 50 million citizens.^2^

The mortality rate associated with cyclone events is higher for women compared to the rate for men in Bangladesh. During cyclone Gorky (1991), women made up 93% of the 140,000 casualties^3^ and almost 83% for cyclone Sidr (2007).^4^ In tropical storm Mahasen (wind speed 63-118 km/h) in 2013, 50 people died, of which 17 were women with the rest being children.^4^

The establishment of cyclone shelters and cyclone preparedness campaigns in the coastal regions have increased cyclone awareness and evacuation adherence in the coastal communities of Bangladesh, which has reduced the death rate from recent cyclone emergencies.^5^ However, in many cases, social isolation caused by a combination of the conservative social setting, people’s social values, and gender-related attitudes resulted in delayed evacuation among women.^6,7^ Women were expected to maintain *purdah* (covering women’s skin and hair and staying away from men other than family members), to look after children and the elderly, and to stay indoors, which narrowed their options to prepare for an imminent cyclone.^7^ Women who evacuated without their husbands reported being sexually assaulted and enduring social humiliation and punishment for not following the guidance of their male counterparts.^8^ These social barriers accompanied by a lack of separate toilet facilities for women, inadequate space for household livestock, unfair (gender-biased) relief distribution, and a scarcity of drinking water, food, and medicines created practical challenges for female evacuees.^4^

Although the extant literature identifies many of the major issues arising in the cyclone shelters that affect women, it does not provide sufficient insight into women’s personal experiences and perspectives on their situation. This paper investigates what it is like to be a female evacuee in the cyclone shelters of Bangladesh to develop an improved understanding of their health and well-being experiences as evacuees.

## Methodology and Methods

Max van Manen’s methodological approach to hermeneutic phenomenology was adopted as the research methodology for the paper.^9,10^ After the requisite ethics approvals from the Social and Behavioral Research Ethics Committee (SBREC) of Flinders University (Adelaide, Australia; Project number: 8197) were in place, a framework was prepared to recruit women to undertake in-depth interviews.

### Selecting the Study Area

**Figure 1.**
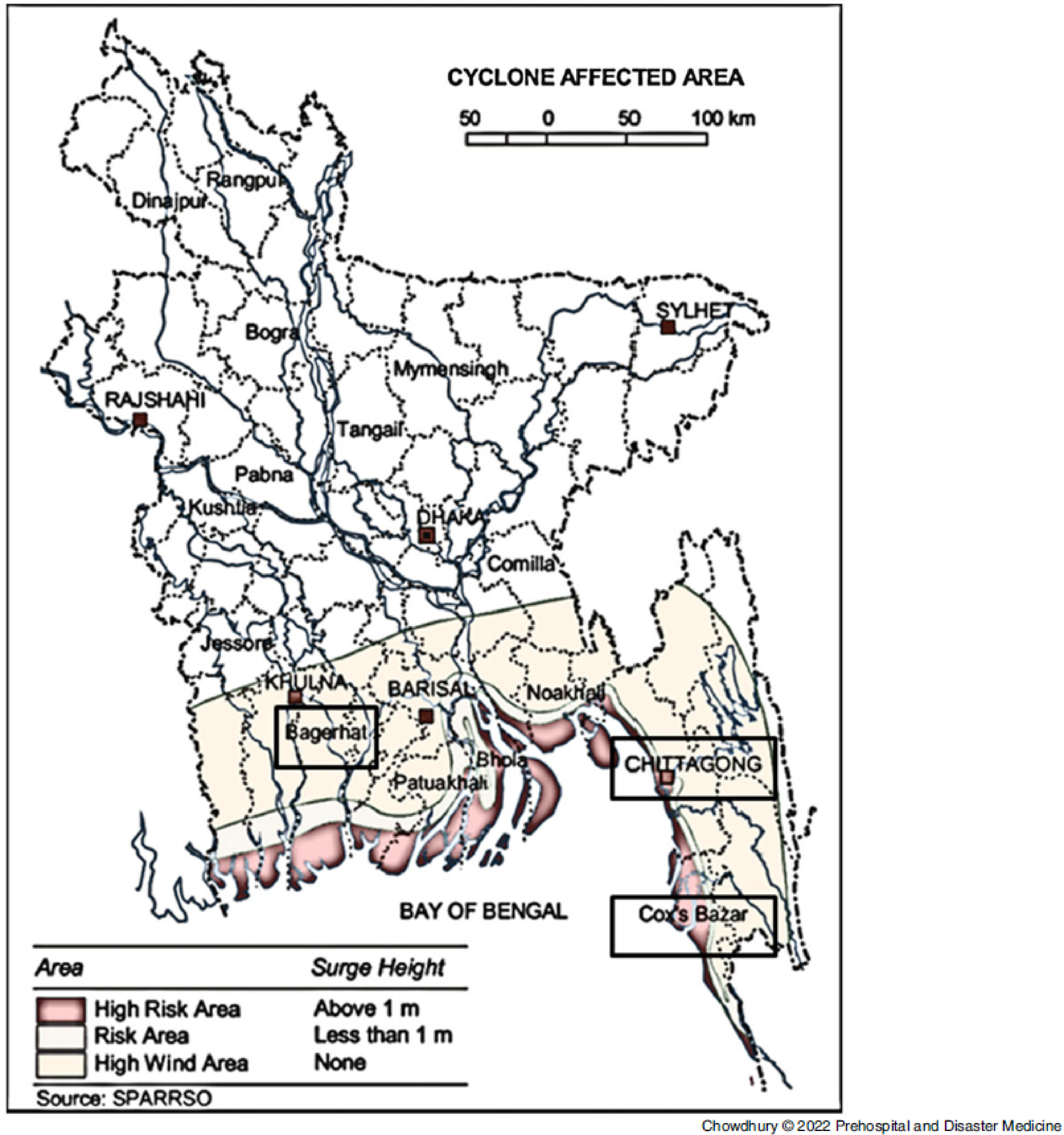
Map of Bangladesh.^12^ Note: The black rectangle boxes depict the districts chosen as study areas.

Among the unions (the smallest rural administrative and local government units in Bangladesh) of the coastal region that are categorized as “highly vulnerable” by the Ministry of Disaster Management and Relief (MoDMR; Dhaka, Bangladesh), the majority are located in the Eastern region, especially in the Chattagram and Cox’s Bazar district, while the remainder are primarily located near the Sundarbans, a mangrove forest in the Ganges delta area in the Western region.^11^ These three districts were selected as the study areas for the research (Figure 1^12^).

Three sub-districts, Banshkhali from Chattagram district, Cox’s Bazar Sadar from Cox’s Bazar, and Rampal from Bagerhat district, were selected as the study areas. The sub-districts had female populations of 219,151; 217,445; and 77,000 and a total of 148; 80; and 11 cyclone shelters, respectively^13,14^ (Table 1). The cyclone shelters were under the management of local government authorities.

**Table 1.** General Overview of the Three Selected Sub-Districts

Name of Sub-District	Total Area (in km^2^)	Population		Women’s Literacy Rate (%)	Marital Status of Women (aged above 10 years)		Number of Cyclone Shelters	Union Health Center	Community Clinic
		Female	Male		Married	Unmarried			
Banshkhali	376.9	219,151	212,011	36.3	60.5%	31.9%	148	3	15
Cox’s Bazar Sadar	228.23	217,445	241,637	47.9	60.0%	34.6%	80	10	26
Rampal	335.45	77,000	78,000	58.0	68.4%	20.1%	11	9	19

### Data Collection

A total of 19 adult women were recruited for interviews who had stayed as evacuees in the shelters at any time over the previous two decades during and after a cyclone, or during a cyclone warning when a cyclone was approaching but did not affect the study area.

Data collection involved two in-depth interviews with each participant. A semi-structured interview schedule was followed for both interviews, which were audio-recorded with the permission of the participants. While conducting the interviews, the researcher (TC) also recorded field notes of the participants’ body language, their facial expressions, and their pauses, silences, and hesitations. These were recorded as personal reflections which then also informed the selection of themes arising from the data analysis.

### Data Analysis

The recorded interviews were transcribed verbatim and translated into English using van Manen’s three-stage approach consisting of holistic, selective, and detailed reading, which was used as an analytical tool to look for meaning within the text and to identify themes from the narratives.^10^ The analysis process involved looking for meaningful words/phrases and meaning within the text, accumulating similar texts/sentences, and generating themes based on the narratives. NVivo-12 (QSR International; Melbourne, Australia) qualitative research software was used to manage the data analysis and to keep track of significant words, phrases, and sentences within the text. The ability of the software to track similar words/phrases and to refer back to the parts of the interviews they came from effectively maintained proper documentation for the hermeneutic circle and enhanced the rigor of generating the themes.

## Results and Discussion

**Figure 2.**
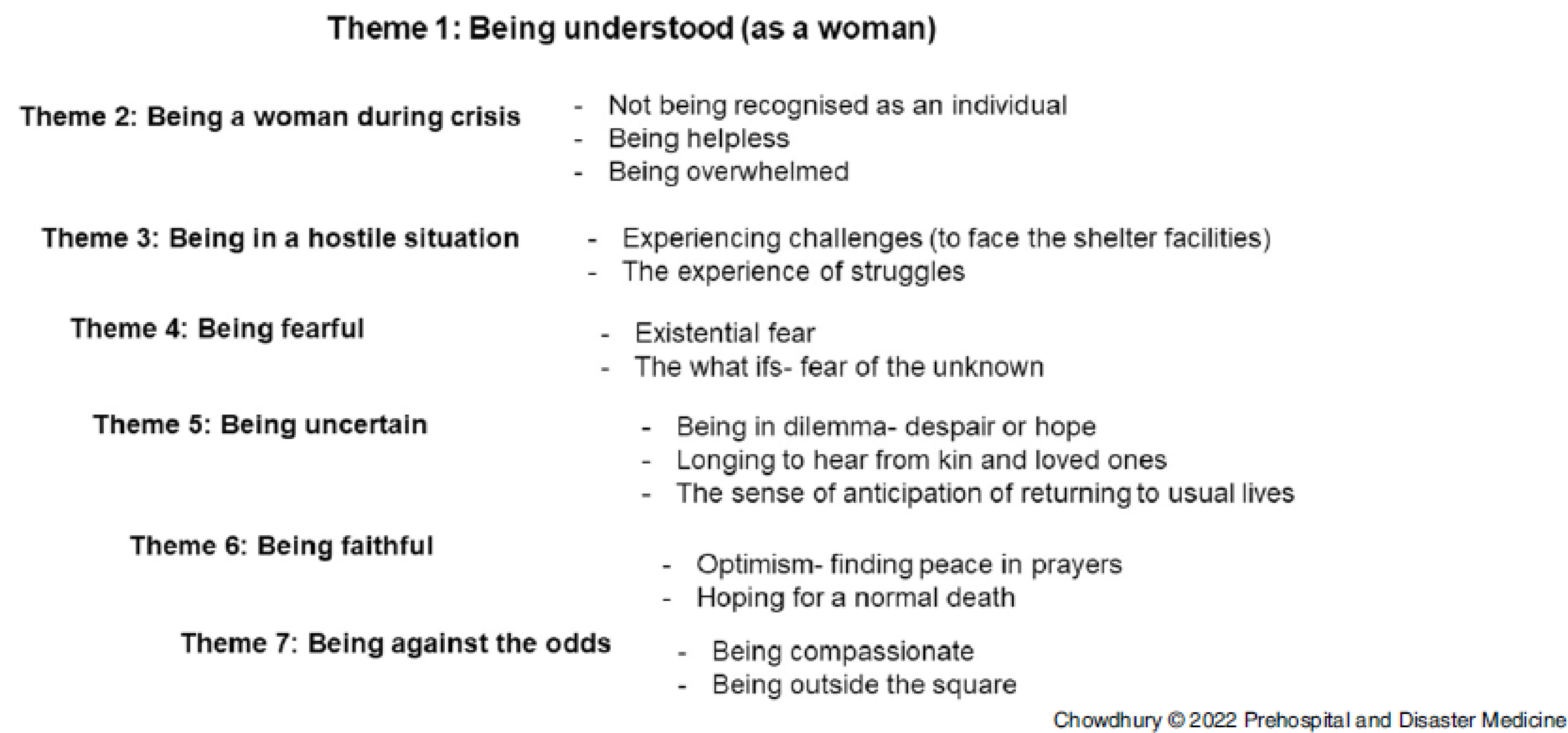
Themes and Sub-Themes Emerging from the Thematic Analysis.

Seven themes and associated sub-themes emerged from the data, revealing the meaning, understandings, and issues the women experienced as evacuees in the cyclone shelters (Figure 2).

### Theme 1: Being Understood (as a Woman)

The first theme, being understood as a woman, surfaced the constant challenges for women in everyday life and appeared as a mode of being, a concept that echoed throughout the narratives. In the areas in which the study was undertaken, it was understood that the honor of the family laid with the virtues of their female family members. Women were taught to stay inside to safeguard their honor and protect their families’ reputations. Notably, these social norms were generally preferred by the women:

I like this rule (staying inside the household) anyway. I do not like talking to unknown people or go outside; I am too shy for that. I like to stay inside, protecting my privacy.

Such social practices also led to women’s financial dependence on men. Even though some of the participants earned money working outside their households, this was not well-accepted in their families and in the community.

The theme of being understood as a woman emerged from the participants’ repeated statements about their hardships and the limitations they faced in their day-to-day lives as women.

The participants frequently discussed their challenges and struggles, which increased dramatically when a cyclone emergency was announced. Being understood as a woman increased their sense of vulnerability in the cyclone shelters, where all evacuees of both genders found themselves cohabiting and in peril due to the imminent disaster.

Because women in the study area were generally not self-sufficient, they felt they lacked needed resources during these emergencies, as one of the participants recollected:

Even if I have seen many storms, I am still afraid of rainy days. Generally, there are no men in the house, just me and my daughter … We get scared, but we cannot leave our household (without men). For us, life (in the emergency) was like a war.

The mindset of perceiving women as the weaker group led to many unfortunate and uncomfortable situations for women in the cyclone shelters, including instances of gender-specific persecution. For example, one participant stated that:

Men mocked us, teased us. Some pushed us while getting up or down of the shelter … There were times in the shelter when I was so ashamed and embarrassed that I could not think of coming out of the shelter.

Women’s actions within their society and the slow evolution of the social settings in the study area resonated with the foundations of Anthony Giddens’ *Structuration Theory*. Giddens introduced the idea of the duality of structure,^15^ suggesting that individuals’ decisions about their respective behaviors within a group create the structure of that group; likewise, the structure of the group encourages and limits the actions of the people within the group. In relation to the themes of the study, being understood as a woman within social situations was adopted and maintained by the individuals from the community and followed and reinforced by the actions of the community. Although Giddens claimed that a person from a particular group could behave differently from other group members in terms of accepting and using the rules and resources of the group, it was apparent in the context of the study area that women were more inclined to follow the rules and maintain the social structures determined by the exercise of their predecessors than using the resources (such as education) available to them. As a result, women’s position within the social structure remained quite static over the years in the study areas, which mirrored their experiences in the cyclone shelters during cyclone emergencies.

### Theme 2: Being a Woman During Crisis

This theme captured the essence of living through a crisis as a woman, answering the question “How does it feel to be a woman in a crisis?” From the participants’ stories, it was evident that being a woman in a crisis was an experience of suffering related to being solely responsible for their children, elderly family members, livestock, and other household possessions, because the male members of the families usually worked outside of the household and sometimes lived and worked in different cities and countries.

One of the women remembered being overwhelmed when she was instructed by her absent husband to save their children, even at the cost of her own life:

He kept reminding me that I must take care of the children. He said that it is okay if I die protecting my kids.

Another participant recounted:

… It was stressful. I got scared. I could not manage myself and not even them (children and the elderly). I had to take responsibility.

A comment from one of the participants “What is the point of a mother to survive if her children are no more?” suggested that the reason behind being protective towards their children might, at least in part, have been a desperate attempt to stay true to the fundamental purpose of their lives. To the women in the context of being understood as a woman, losing their child/children meant losing the purpose of their lives. In the past, women in the community were shamed and blamed when their children died by accident, and the women feared not only losing a child, but also the traumatic experience of being considered unsuccessful as a mother and a woman.

In this theme, the participants were unified in their understanding that during a crisis, women’s multi-tiered roles of protectors, carers, and hoarders resonated with Enarson’s theory on women’s increased physical and emotional labor during different phases of a disaster^16^ and their increased exposure to short- and long-term psychological disorders.^17,18^ The findings of the theme also aligned with women’s experiences during the recurrent floods in Manila in the Philippines where 77.9% of the female participants were responsible for organizing food for the family, and 55.9% were carers for sick family members, which increased their psychological distress.^19^ Similar to the unfair situational expectations of these Filipino women, the urgency of fulfilling women’s responsibilities and the frustration of not meeting societal expectations left the participants feeling helpless and overwhelmed in the cyclone shelters.

### Theme 3: Being in a Hostile Situation

The limitations of the shelters had an impact on every evacuee, regardless of their gender; however, the women tended to suffer more in the shelter because of being positioned as a woman.

Most of the women were concerned about being exposed and suffering humiliation and embarrassment from their fellow evacuees and could not get past their shyness to make use of the toilets, talk to other people, and find suitable space, water, or food. As one of the participants recalled:

I could not go to the shelter toilet. I ruined (urinated on) my clothes.

She also remembered:

It was very hard for me to climb up the stairs. I have joint pain. Once I fell and fractured myself.

The women described a range of experiences that affected their physical and mental health and negatively affected their lives. For example, a newly-wed participant shared her trauma of having children after having to watch her sister give birth in one of the cyclone shelters:

I will remember this until my death. We were awake all night long. It became a matter of life and death … (after the delivery). My sister could not move. We took her to the home just like a dead body. She had no sense all night long … I was young and unmarried then, I did not know the way children were born, and I still feel nervous when I reminiscence that incident.

Another evacuee described her experience of living in the shelter a few days after childbirth:

… My stitches (from childbirth) were taking time to heal, I was weak … I sat on the floor for hours, went to urinate just at the last moment. These caused too much pain and discomfort, in fact, because of all these I needed to restitch my wound.

This theme revealed that women found themselves in a disadvantaged position in the shelter, which was similar to the experiences of women across the globe. For example, women from Sri Lanka and India were reported to have faced physical and mental abuse from sexual assault, violence, and gender-bias while living as evacuees after the 2004 Indian Ocean tsunami,^20,21^ which were frequently shared experiences for the participants in this study. One of the participants remembered her sister giving birth inside the shelter with no medical assistance, which is similar to the situation of female evacuees from Pakistan following the Hindu Kush earthquake (2015).^22^ The associated anxiety and long-term trauma of pregnant and lactating women and women who witnessed these struggles are also reflected in the literature on women’s experiences in the evacuation centers established for the Nepal earthquake in 2015.^23^

The unique personal reflections highlight the diversity of experiences among the female evacuees, which affected both their short- and long-term physical and mental health.

### Theme 4: Being Fearful

The women had an extreme fear of the prospect of cyclones in coming years, even after having survived several in the past. These women had witnessed the level of destruction, loss, and damage a cyclone could cause, and in some cases, had experienced personal loss which had left a psychological impact. Some participants confessed to experiencing on-going trauma because of particular incidents in the shelters, including injuries/abuse, and/or seeing the corpses of cyclone victims. One participant recounted:

I still remember the flood of 1991. Two of my kids died by that time …

As another participant explained:

I feel afraid and restless about how to save my life. Then I worry about my cattle, and where should I keep them.

Another participant remembered having a nervous breakdown at the shelter as a result of being frightened about being assaulted or embarrassed by male evacuees and thereby losing her honor:

Everything was frightening. I was afraid and wondered could I even return home with all my honor!

Cvetković, Öcal, and Ivanov^24^ suggested that people are likely to experience anxiety associated with security in the shelters, post-disaster violence, forced migration, hostility, and discrimination in the post-disaster phase. This explains women’s subsequent mental stress and fear described in the theme being fearful, which mirrors the innate human reaction of fear during catastrophic events, thus revealing the connection between the feeling of fear and the participants’ reality.

### Theme 5: Being Uncertain

Although most of the women admitted being in similar situations on several previous occasions and could comprehend the upcoming emergencies, they could not stop worrying about experiencing something unexpected and unfortunate, which led to uncertainty and deeply affected their sense of well-being in the shelter. As one of the participants stated:

… Nothing was certain … What would I do if something happens to my kids? … What would they do if something happens to me?

The women voiced their concerns for the safety and well-being of their significant others who were away and expressed feeling hesitant and vulnerable about not being able to contact their husbands. A participant whose parents and other family members lived in different villages near the coastal area and whose husband was living abroad explained:

I could not contact my husband, my relatives, and my parents, because the cell phone had no charge. I was worried about them and felt sick from all the stress.

While sharing their experiences, the participants remembered their urge to return home. In most cases, they found their homes damaged or destroyed and their post-storm experiences included laborious and hectic rebuilding. Yet, a sense of relief was palpable in their voices, and this was reflected in their body language as they described their experiences of returning to their homes.

Stein and Stein^25^ argued that sometimes the probabilities of outcomes from disasters remain unknown, or less known, because of the multiple possibilities and diversities of past experiences which result in deep levels of uncertainty. This psychological feature explains the sense of uncertainty among the participants in the shelter, even after acknowledging being safe in the shelter. Given past devastation and struggles from cyclones, the women felt the threat of facing unforeseen and poorly understood impacts that they had not experienced in the past during their stay in the shelters.

### Theme 6: Being Faithful

This theme addresses the religious beliefs and spiritual faith of the participants that shaped their being and influenced the frames of reference they used to organize and understand their experiences, and specifically, their experiences of living through cyclones in the shelters.

The participants described praying constantly to the Almighty, both individually and communally, which helped them to remain optimistic and gave them a sense of security. The women had developed a fatalistic outlook tending to leave everything to a greater power and accepting both their good and bad experiences as the will of the Almighty. This mindset tended to moderate their reactions to the impediments, injustice, and discrimination they had suffered throughout their lives and their unwavering faith had helped them to pass through the troublesome and painful situations and challenges of the cyclone shelters. Amidst all the negativities that the participants had battled through, it was often their faith that gave them some relief from stress and anxiety and restored their mental health to some extent. As one participant remembered:

… I felt scared, but then (I told myself) Allah is here to protect us.

Hoping for a normal death was also expressed in the narratives of the participants, which appeared as a powerful concept among the women. During the cyclone and inside the shelters, women faced life-or-death situations that led many of them to wonder about their deaths. The women from the study area witnessed many deaths and casualties from the cyclones and developed a fear of dying by drowning and experiencing suffering from injuries and trauma. One participant stated:

… I mean, Allah can take us anytime. I would die a painful death if I die in the storm. This makes me upset. My life is full of struggle, I want a peaceful death.

Contrary to this, based on their faith, some participants delayed going to the shelter or did not evacuate at all:

Allah will decide people’s death. I am just going to put all my trust in Him. He knows the best.

While the women’s anticipation of a normal death defined the fear and trauma associated with past cyclones, it also reflected the depth of their faith and captured how their faith interacted with their self-worth, optimism, and sense of being, which consequently influenced their state of well-being in the shelters.

The theme is consistent with Abbott and White’s^26^ argument that in the wake of a disaster event, faith provides people with a framework through which to comprehend the shock while also sustaining patience, peace, and a concern for others. In the shelter when women were constantly challenged physically and mentally, faith gave them comfort and became the most effective way to restore their sanity from the on-going predicament.

### Theme 7: Being Against the Odds

This theme highlights the significant role played by the female evacuees in the shelters in being resilient and resourceful to provide for their own primary health needs and those of their fellow evacuees.

Some of the women remembered arriving at the shelter prepared with adequate food supplies, drinking water, medicines, and other necessities because they had acted promptly after the emergency announcements instead of delaying. Some participants shared their food, water, and medicines with fellow evacuees and offered help to clean and maintain hygiene at the shelter. However, such actions sometimes worsened the experiences of the women who rendered such selfless assistance. The motivation of these women was often misunderstood and they received harsh rejection and rude comments from others, which discouraged them from continuing to help out in the shelters. The disappointment and dismay they felt from the negative attitudes were reflected in their stories. One woman stated:

Still, I try to help others maintaining my self-respect.

One of the participants recalled her experience of voluntarily taking a leadership role in the shelter where she gathered like-minded people from the community and worked for the evacuees at the shelter by arranging basic requirements including food, drinking water, medicines, and sanitary supplies; by identifying sick and disabled people; by allocating separate space for pregnant and lactating mothers; by identifying the immediate needs of the evacuees; and by communicating with local NGO authorities and government entities.

The examples of compassion, initiation, and innovation to facilitate health and well-being needs for themselves and other evacuees proved the women’s capacity to act as resourceful individuals instead of only being victims in the shelters.

The theme highlighted the women’s capacity to be resourceful, take initiative, and cooperate with each other in emergencies despite their disadvantaged positions and circumstances in the cyclone shelters. This notion of compassionate action(s), often beyond their comfort zone, and reaching out to fellow evacuees can be explained through Aldrich and Meyer’s^27^ idea of bonding social capital that influences neighbors to cooperate and, at times, to become the first responder in emergencies.

### Making Sense of Women’s Experiences

The themes of this study uncovered the meanings and understandings associated with women’s experiences and revealed a number of health challenges including physical injuries and trauma, anxiety, and panic attacks leading to subsequent posttraumatic stress disorder (PTSD), genito-urinary infections, and post-natal health issues, which affected female evacuees’ overall well-being in the shelter and later in their lives. The findings of the study signify how women’s societal position and cultural and spiritual mindset influenced their sense of well-being. While being understood as a woman within the coastal community instigated various health challenges and examples of mental trauma, being faithful provided them with the ability to remain optimistic and help others in need. However, women’s position in society was supported and practiced by the women themselves, which underlines the importance of acknowledging their beliefs while planning improvements to their situation as evacuees. The last theme of being against the odds demonstrated the women’s capacity to venture outside the square without compromising their socio-cultural settings and acting as first responders for other evacuees in health emergencies. With proper training and education, these women would have the potential to assess and stabilize injury and trauma victims in the shelters and to identify and provide primary care to women with reproductive and mental health issues with limited and/or no outside assistance.

## Limitations

The lived-experiences of the specific cohort revealed in this research may not reflect the experiences of other woman, as the experiences are somewhat specific to this group of women in this particular context. However, it is likely that the themes identified are shared across many women who have been evacuated to cyclone shelters in Bangladesh.

## Conclusion

This study gives the women from the study area a voice to narrate their own perspectives on what they consider to be challenges to their health and well-being. This discovery frames the necessity of using local human resources to improve women’s health and well-being experiences in the shelters without contravening their socio-cultural norms and religious ideology.
